# Exploring the health‐related decision‐making experiences of people with chronic kidney disease and their caregivers: A qualitative study

**DOI:** 10.1111/hex.13907

**Published:** 2023-11-05

**Authors:** Shena Gazaway, Orlando Gutierrez, Rachel Wells, Tamara Nix‐Parker, Claretha Lyas, Shawona Daniel, Katina Lang‐Lindsey, Tara Bryant, Richard Knight, James N. Odom

**Affiliations:** ^1^ Division Family, Commuity, & Health Systems, School of Nursing University of Alabama at Birmingham Birmingham Alabama USA; ^2^ Center for Palliative and Supportive Care University of Alabama at Birmingham Birmingham Alabama USA; ^3^ Nephrology Training and Research Center University of Alabama at Birmingham Birmingham Alabama USA; ^4^ Division of Nephrology, Heersink School of Medicine University of Alabama at Birmingham Birmingham Alabama USA; ^5^ Division‐Acute, Chronic & Continuing Care, School of Nursing University of Alabama at Birmingham Birmingham Alabama USA; ^6^ School of Nursing University of Alabama at Birmingham Birmingham Alabama USA; ^7^ Department of Social Work, Psychology and Counseling Alabama A&M University Huntsville Alabama USA; ^8^ Viva Health Inc. Birmingham Alabama USA; ^9^ American Association of Kidney Patients Tampa Florida USA

**Keywords:** community‐involved methods, decision‐making, qualitative methods

## Abstract

**Background:**

This study aimed to explore the decision‐making experience of patients with chronic kidney disease (CKD) and their caregivers.

**Methods:**

This was a qualitative descriptive study of the decision‐making experiences of individuals with stage 3—end‐stage CKD and their family caregivers. One‐on‐one, semistructured interviews were conducted using a guide developed and approved by a community advisory group. Data were analyzed using thematic analysis.

**Results:**

Three themes were identified: (1) decisions triggered by declining health and broad in scope, (2) challenges to decision‐making and (3) factors influencing decision‐making. Participants' experiences with health‐related decision‐making demonstrated that decisions were triggered when health declined. Yet, decisions that impact disease progression were being made in stage 3. Decision‐making was made difficult due to lack of information, complex co‐morbidities, and poor resource utilization. However, the structure and nature of the medical appointment, supportive caregivers, and resources served to remove challenges.

**Conclusion:**

Decision‐support interventions must train patients and caregivers to be empowered participants in answer‐seeking behaviours upstream of advanced illness.

**Public Contributions:**

This work was conducted in full collaboration with a community advisory board consisting of patients with CKD, caregivers and clinicians. These members are noted in the acknowledgement section, and those who worked with the team to develop the interview guide, study protocols, and manuscript preparation are included as authors. As part of their role, advisory members met monthly, providing input on recruitment, study progress, inclusion of diverse voices and added relevance to study findings.

## 1 INTRODUCTION

Approximately 37 million US adults live with chronic kidney disease (CKD). A total of 786,000 people will advance to end‐stage renal disease.^1^ CKD is progressive and categorized into stages (1–5), diagnosed based on multiple laboratory tests, with the hallmark test being the glomerular filtration rate, which is a measure of kidney function.^2^ Within the confines of this article, we will focus on stages 3–5, as many adults rarely are aware they have the disease until stage 3 when referral to specialty nephrology care is sought, but still, for many, diagnosis of CKD may occur in an emergency setting.^3^ Stage 3 CKD, like stages 1–2, is often asymptomatic, and treatment focuses on treating the underlying cause of disease, such as—hypertension or diabetes.^4^, ^5^ Thus, lifestyle and treatment recommendations focus on controlling the disease process; this is another reason specialty nephrology care may not be sought in stage 3 because the focus has not yet turned to preserve kidney function.^4^

Individuals with stage 4 CKD may experience fatigue, swelling, changes in urination patterns and anaemia. In addition, there may also be concerns with control of their underlying conditions, which can increase their risk of complications, including worsening cardiovascular outcomes.^4^, ^5^ Quality of life for these individuals can worsen as treatment focuses on tighter management of disease processes, which could mean stricter dietary management, increased dosages of medications, newer medications and increased fatigue or medication side effects impacting daily life, thereby limiting individuals' ability to fully participate in work or activities that bring joy or fulfilment.^5^, ^6^ In stage 4, individuals may begin discussing options for renal replacement therapy, such as dialysis types (in centre or home, peritoneal dialysis), or renal transplantation.

Stage 5 is characterized by severe kidney function loss, leading to an accumulation of waste products within the body because the kidneys are ceasing to function as the body's filtration system. Symptoms include extreme fatigue, nausea, vomiting, itching, muscle cramps, cognitive impairment and fluid retention, leading to massive swelling.^5^ Quality of life in stage 5 is impacted significantly due to both disease progression and the chosen treatment option, especially if haemodialyses is the treatment of choice.^5^, ^6^ As a treatment, haemodialyses is time‐consuming and carries its own unique symptoms, stressors and complications. In‐centre haemodialyses are done 2–3 days a week, over multiple hours and patients complain of fatigue, pain and other distressing symptoms after treatment, including fluid imbalances.^7^, ^8^ Those who conduct in‐home haemodialyses or peritoneal dialysis often do so on a daily schedule, usually dialyzing over longer periods at night, which is not as demanding on the body but still has limitations to life and other stressors related to daily activities. Even choosing transplantation includes unique challenges, risks and procedures regarding organ matching, selection and possible rejection, and the need for lifelong medication.^9^, ^10^

Family caregiver involvement is often introduced in the literature when pertaining to the care of individuals with stage 5 CKD; sometimes, caregiving is addressed in stage 4, depending on the complexity of the underlying disease or the patient's age. Family caregivers are the essential unseen ‘workforce’ of the medical community. Family caregivers assist their loved ones with activities of daily living and aid in managing medical/nursing tasks daily.^11^ Like cancer and other chronic conditions, family caregivers are relied upon in advanced CKD for more than just daily assistance; they support managing medications and symptoms, care coordination, transportation and household management. Caregivers perform caregiving duties 10–26 h per week or more with little to no compensation, and many have little to no support outside of the dyad.^11^, ^12^ In addition to supporting the direct medical needs of the seriously ill, caregivers are known to be present when their loved one is hospitalized, which can be a frequent occurrence in stage 5 disease, assisting with health‐related decision‐making; yet, despite their reporting active engagement in the decision‐making process, a quarter describe only being involved by healthcare providers some of the time.^12^, ^13^, ^14^, ^15^, ^16^ Examples of involvement include engagement with decisions about renal replacement treatment choice, disease management, when to seek emergency care, how to pay for care, obtaining additional help and end‐of‐life care.^14^, ^15^, ^17^, ^18^ As decision support partners, caregivers help patients make decisions by seeking information, communicating, assisting patients to weigh risks and benefits and understand their options, eliciting patients' values, planning for future decisions and implementing decisions.^9^, ^19^, ^20^, ^21^, ^22^, ^23^ To signify the impact of a caregiver's influence, we highlight that studies have demonstrated that family and friends' approval or disapproval influence engagement with preventative cancer screenings and organ donor registration for patients, which signifies the potential weight these individuals have on health‐related decision‐making.^24^, ^25^

One aspect of health‐related decision‐making is treatment decision‐making. Treatment decision‐making when stage 4 and 5 CKD is diagnosed (considered advanced CKD) starts with understanding complex health information, such as medication options, nutritional guidelines, the underlying pathophysiology of CKD and its progression and consideration of renal replacement options (discussed above). These factors are imperative to understand in advanced CKD due to an intense change in medication regimen and dietary restrictions that are often needed, compared to earlier stage disease, to delay worsening disease. When speaking from naturalist decision‐making theory, these actions would mean that the patient must have the ability for interoception (accurate self‐perceptions) and exteroception (accurate perception of the outside world).^26^ Additionally, CKD patients must often coordinate with others to make decisions and frequently are unaware of how to partner with clinicians when making treatment decisions.

The naturalistic decision‐making framework emphasizes the importance of understanding how decisions are made in the real world, allowing for a more complex picture of decision‐making under dynamic conditions.^27^ When applied to chronic illness decision‐making, the process reflects a ‘whole mind’, naturalistic approach encompassing real‐life contexts where patients discover and resolve their values and goals.^28^ The naturalistic decision‐making framework has been applied to heart failure self‐maintenance literature when exploring decisions to engage in self‐care activities. As a naturalistic decision, engaging in self‐care is a decision that has meaning and is familiar to the patient, thus is one that they have engaged with and would be natural to continue doing within the context of self‐management of their heart failure.^29^ When applied to CKD, the literature demonstrates that many patients desire detailed information about their disease and prognosis^30^; clinicians often report difficulties in sharing complex prognostic information that can lead to misinformation to maintain hope.^31^, ^32^ The consequences of these treatment decision‐making practices and trends in CKD leave patients and caregivers underinformed, potentially resulting in missed opportunities for them to take greater ownership of their disease management and be taken by surprise when suddenly faced with decisions about their renal replacement therapy choice.^28^, ^33^

Given the scarcity of literature describing health‐related decision‐making before initiating renal replacement therapy,^34^, ^35^, ^36^, ^37^, ^38^, ^39^ we undertook a study to qualitatively describe the needs, challenges and experiences of patients with CKD and their family caregivers when making health‐related decisions from stage 3—end‐stage disease. In capturing decision‐making within this wide range of disease, it is imperative to understand the decisions being made, what triggers major decision discussions, and the unique barriers to decision‐making in each stage. In doing so, these results inform the development of an early, dyadic decisional‐support intervention to enhance dyadic coping, decrease decision conflict and stress and enhance quality of life.

## 2 METHODS

### 2.1 Study design

Following the Consolidated Criteria for Reporting Qualitative Research^40^ guidelines, a qualitative descriptive study^41^, ^42^ aimed at exploring the needs, challenges and experiences of patients with CKD from stage 3—end‐stage and their family caregivers when making health‐related decisions was conducted from November 2021 to December 2022. We engaged a community advisory group of six individuals, including patients, caregivers and clinicians, to provide guidance and oversight in developing the interview guide, implementing recruitment procedures and enhancing the meaningfulness of the final analysis. The Institutional Review Board at the University of Alabama at Birmingham approved this study.

### 2.2 Setting and participants

Participants were recruited from two partnering nephrology clinics at a large academic health science centre in the US Southeast. Patient eligibility criteria included: (1) age 18 or older and (2) a diagnosis of stage 3—end‐stage CKD. Patients were excluded if: (1) they had uncorrected hearing loss, (2) could not read and understand English, (3) had a documented dementia diagnosis or (4) had an untreated major mental health disorder (e.g., major depressive syndrome or schizophrenia). After electronic medical record screening and nephrologist approval, patients were mailed a recruitment letter and a copy of their informed consent. Patients were then contacted by phone and invited to participate; verbal consent was collected. Consenting patients were asked if they had someone whom they spoke to about their healthcare decisions or who their caregiver was.

Patients with no caregiver or who felt that the caregiver would not be interested in participating were not excluded from the study, but those who said their caregiver would be interested either shared that individual's contact information or directed the caregiver to contact us. Caregiver eligibility criteria included: (1) age 18 or older; (2) self‐endorsing or identified as ‘a relative, friend, or partner that has a close relationship with you and who assists you with your medical decisions and who may or may not live in the same residence as you; who is not paid for their help’; (3) caring for a patient with CKD and (4) ability to speak and read English. Exclusion criteria included: (1) self‐reported untreated mental illness; (2) dementia; (3) active suicidal ideation; (4) uncorrected hearing loss or (5) active substance abuse. Caregivers were spoken to via phone, and the informed consent form was read to the caregiver over the phone, with a copy mailed to their home if they verbally consented to participate. All participants were offered $40 as an incentive for participation.

### 2.3 Data collection

A semistructured interview guide was developed after a review of the literature and finalized by advisory group members. Guide topics included health‐related decision‐making experiences and challenges, what helped decision‐making, and how caregivers assisted decision‐making (see Table 1 for a complete guide).

**Table 1:** Interview guide.

For persons with chronic kidney disease	For caregivers only	
Briefly and without going into detail, tell me about your illness and the treatment you are currently undergoing.	Briefly and without going into detail, tell me about the care you've been providing to your _______________.	
What decisional points have you faced during your illness? Can you describe them or label them?	What decisional points have you assisted your ___________ with during their illness. Can you describe them?	
*General questions for both*		
What do you think are the greatest challenges faced by people with Chronic Kidney Disease when they have to make health‐related medical decisions?		
What/who has helped you the most with health‐related decision making? What support do these resources/people provide?		

For persons with chronic kidney disease	For caregivers only	
What kinds of conversations have you had or are you having now about your illness and its impact on your life in the future? Who are you having those conversations with?	What kind of conversations have you had or are you having with your ____________ about their illness and its impact on their life in the future?	
*General questions for both*		
What healthcare decisions do family caregivers help care recipients with? [if people don't feel like they've made choices, ask did you feel like you had a choice]		
What things make it difficult for family caregivers to help the individuals they care for make healthcare decisions?		
What information or assistance would be helpful to family caregivers as they help individuals, they care for make healthcare decisions?		
What are the biggest decisions that you think family caregivers themselves will have to make during their care recipient's serious illness?		
What information of assistance would be helpful to them in preparing for these decisions?		
Are there any closing thoughts on health‐related decision making that you would like to share with me?		

Interviews were audio recorded and transcribed verbatim by a professional transcription service (Landmark Associates Inc.).

### 2.4 Data analysis

Transcripts were entered into NVivo© qualitative analysis software. Thematic analysis was used to code and identify themes deductively from the data systematically.^42^, ^43^ Data analysis occurred as an iterative process as interviews were being conducted. Saturation was achieved at interview 22; eight additional interviews were added to enhance the sample's diversity and collect more caregiver voices.

The study team engaged in the following activities throughout the study timeframe to enhance the trustworthiness of the analysis provided. Credibility was enhanced by the methods described here. Method 1 was analyst triangulation; transcripts were first independently analyzed by S. G. and T. N.‐P. Both had training in qualitative data analysis and met regularly throughout the analysis to discuss preliminary and emerging codes, revisit and revise the coding scheme and finalize themes. Method 2 was prolonged engagement; the authors presented the themes to community advisory board members once the analysis concluded. Although each theme was presented individually, with supporting codes and quotes from the data, members were also given hard copies of the handouts. Members agreed that the presented themes were consistent with their experiences with decision‐making as a CKD patient, caregiver or clinician. Confirmability was enhanced throughout the process of reflexivity and creating an audit trail; the first author took notes throughout the literature review, interviews and analysis to assist with interpretation. In addition, this act assisted her in bracketing her own healthcare and firsthand experiences as a CKD caregiver.

## 3 RESULTS

### 3.1 Participant demographics

Thirty interviews were completed with 22 patients and 8 caregivers. Interviews ranged from 15 to 60 min, with the majority lasting about 40 min. All demographics were self‐reported data, except CKD stage, which was part of the eligibility criteria. Most of the patient participants were White (54.5%), female (77.3%), had stage 4 disease or higher (54.6%) (representing advanced kidney disease) and multimorbidity (59.1%), including hypertension and diabetes (Table 2).

**Table 2:** Participant characteristics.

Characteristics	Overall (%), *N* = 30	
	Patient (*n* = 22)	Caregiver (*n* = 8)
Sex		
Female	17 (77.3%)	4 (50%)
Male	5 (22.7%)	4 (50%)
Age		
21–30	0 (0%)	0 (0%)
31–40	1 (4.5%)	0 (0%)
41–50	2 (9.1%)	0 (0%)
51–60	4 (18.2%)	3 (37.5%)
61–70	9 (40.9%)	3 (37.5%)
71–80	6 (27.3%)	2 (25%)
Race		
Black	10 (45.5%)	4 (50%)
White	12 (54.5%)	4 (50%)
Religious affiliation		
Yes	20 (90.9%)	8 (100%)
No	2 (9.1%)	0 (0%)
Marital status		
Married	12 (54.5%)	5 (62.5%)
Divorced	4 (18.2%)	1 (12.5%)
Single	4 (18.2%)	1 (12.5%)
Widowed	2 (9.1%)	1 (12.5%)
CKD stage		
3	10 (45.5%)	–
4	6 (27.3%)	–
5	4 (18.2%)	–
End‐stage dialysis	2 (9.1%)	–
Employment status		
Fulltime	6 (27.3%)	1 (12.5%)
Parttime	1 (4.5%)	2 (25%)
Retired	9 (40.9)	3 (37.5%)
Disabled	2 (9.1%)	0 (0%)
Unemployed	2 (9.1%)	2 (25%)
Self‐employed	2 (9.1%)	0 (0%)
Education		
Less high school	1 (4.5%)	0 (0%)
High school/GED	6 (27.3%)	0 (0%)
Some college	5 (22.7%)	4 (50%)
Associate degrEe	2 (9.1%)	2 (25%)
Bachelor's degree	5 (22.7%)	2 (25%)
Master's degree	2 (9.1%)	0 (0%)
No answer	1 (4.5%)	0 (0%)
Insurance		
Private	10 (45.5%)	5 (62.5%)
Medicaid	1 (4.5%)	0 (0%)
Medicare	5 (22.7%)	2 (25%)
Medicare + Medicaid	1 (4.5%)	0
Private + Medicare	4 (18.2%)	2 (25%)
No answer	1 (4.5%)	0
Internet access		
Yes	21 (95.%)	7 (87.5%)
No	1 (4.5%)	1 (12.5%)
Internet comfort		
Yes	12 (54.5%)	6 (75%)
No	10 (45.5%)	2 (25%)
Relationship to patient		
Spouse	–	6 (75%)
Sibling	–	2 (25%)
Lives with patient		
Yes	–	6 (75%)
No	–	2 (25%)
Started caregiving		
10 years or more	–	4 (50%)
Between 9 and 5 years	–	1 (12.5%)
Between 4 and 1 years	–	1 (12.5%)
Less than 1 year	–	0 (0%)
No answer	–	2 (25%)

Abbreviations: CKD, chronic kidney disease; GED, General Educational Development.

The majority were over 60 (68.2%; *M*_age_ = 63.4; range = 31–80). Patient participants were mostly retired (40.9%), had some college education or higher (63.6%) and had insurance through private carriers (45.5%). The eight caregivers interviewed were evenly split White and Black, male, and female. Caregivers were over age 60 (62.5%; *M*_age_ 64.9; range = 51–80). All caregivers had attended some or graduated from college, and most had private insurance (62.5%), were the spouse of a CKD patient (75%) and had been providing decision support for more than 10 years (50%). In addition, most patients and caregiver participants identified as religiously affiliated (patient 90.9%; caregiver 100%) and had access to and were comfortable using the internet.

### 3.2 Themes

Three themes emerged: (1) decisions triggered by declining health and were broad in scope, (2) challenges to decision‐making and (3) factors influencing decision‐making. These findings were common among both patient and caregiver participants. Examples of participant quotations to illustrate each theme are shown in Table 3.

**Table 3:** Quotations illustrating themes and subthemes.

Decisions triggered by declining health and were broad in scope
I'd go in, and he'd tell me, you know, well, you know, your‐your disease is getting a little worse, you know, but I was getting no numbers. And I didn't understand the disease, and I didn't have any education on it, and no one really told me. So I didn't know anything until my numbers got to where what, 19, and at 19, I said, ‘Well, doing nothing is just not an option for me’. (∼Patient Participant—female)
So I was tryin' to maintain as long as I could, but as my function became lower and lower, my doctor [audio cuts out 01:00] to a nephrologist, to use—have three just so I could gather as much information as possible. They were all sayin,’ ‘Your transplant is on the horizon. Be lookin' into ‘that’. (∼Patient Participant—male)

Challenges to decision‐making
*Not enough information about the pathophysiological process of CKD and available resources*
I was going there every three months to see him, but we would just—you know, I'd go in, and he'd tell me, you know, well, you know, your‐your disease is getting a little worse, you know, but I was getting no numbers. And I didn't understand the disease, and I didn't have any education on it, and no one really told me. So, I didn't know anything until my numbers got to where what, 19, and at 19, I said, ‘Well, doing nothing is just not an option for me’. (∼Patient Participant—female)
Well, at least give me some knowledge, that's what I'm saying. Lack of knowledge is one of the key things, you talk about renal failure, and you tell people about the onset and you tell ‘em about some of the signs and the symptoms, but once you're in it, what do you do? (∼Patient Participant–female)
What information. You know, she went through this, class thing, and she still don't do what she's supposed to do on it. But I guess if she'd had some more programs that would inform her of what could happen if she do this, that, or the other. (∼Caregiver Participant—male)
*Co‐morbidities made it difficult to prioritize CKD issues*
And, uh, you know, I would just ask them for something for pain. And that's when they told me that. Uh, Dr. X never told me. You know, they was, uh, the doctor told me that, uh, my primary doctor was saying that he‐he couldn't give me anything strong because of my, uh, my kidneys. (∼Patient Participant—female)
Um, and I've‐I've got—I've not only got kidney disease, but I've got this heart disease, and I've got a hernia that needs to be fixed, um, and a wisdom tooth that needs to come out. Um, but they would not do the hernia surgery because of my heart. (∼Patient Participant—male)

Factors influencing decision‐making
*Caregivers*
He's 100 percent on it with me, more so than my children. Um, um, he's with me all the time, and, uh, he‐he‐he helps me. He goes to the classes with me. He sits with me. Um, all of my‐my doctor's appointment, he makes sure I'm there. He makes sure that, uh, I'm on time. You know, it's very rarely I miss appointments, you know. He's 100 percent with me on this. (∼Patient Participant—female)
We lose patience because sometime I feel like I'm losing it, and sometime I feel like I'm‐I'm not gonna make it, you know. And then between that and‐and my partner, he put—he let me know, too that, you know, God got this for you. You know, you can't lose the faith. You know, your doctors are good. They've been taking care of you. And‐and like I say, I'm blessed from 40‐something to 70 without dialysis or anything like that, that it's nothing but the grace of God and my doctors. Uh, well, he—his team ‘cause that's who He got to‐to help me, to keep me where I'm at today and my partner, and ‘cause I couldn't do it without them. (∼Patient Participant—female)
That's what help me. Whenever I go—you know, listenin’ to what the doctor says at our next appointment, and then if they—somebody don't follow through, then I can call back to see. ‘Okay, well, this didn't happen. What do we need to do now? Because this don't seem to be working’. (∼Caregiver Participant—female)
*Decisions in the medical appointment context*
I take a notebook to my doctor's appointment, and any time I have any questions, um, they're there to answer them. And also, we can—I can go on the patient portal and send them a message, and they will send that—you know, whatever's concernin’ me an answer. (∼Patient Participant—female)
But Dr. X said, you know, really takes the time to explain to us, you know, what's going on and, uh, keeping up with my test results and that kind of thing. (∼Patient Participant—male)
I feel very close to our doctor at UAB, the nephrologist. She's very caring and concerned, and, um, very supportive of us. I feel, you know, relying on her input along with other doctors also, his heart doctor, and just family. (∼Caregiver Participant—male)
*Resources*
And, uh, also we have support groups. I've gone to, uh, you know, the groups where we all have kidney failure, and we, you know, try to talk about recipes and different things of that nature. (∼Patient Participant—female)
Uh, the people that I met, I‐I made some relationships with people at UAB when I would go in and do the video. I did that twice, and I did make contact with a few people that I could talk to that were goin’ through the same thing I was. (∼Patient Participant—male)
Um, UAB's website. Um, check out the information about the doctors there and what procedures they do. And, um, just other internet sites also. Um, I am a part of a group, um, um, kidney transplant group. (∼Caregiver Participant—female)

Abbreviations: CKD, chronic kidney disease; UAB, The University of Alabama at Birmingham.

#### 3.2.1 Decisions triggered by declining health and were broad in scope

Decisions were often triggered by declining kidney function necessitating decisions about renal replacement therapy, or the patient needing to make lifestyle changes or take medications to slow progression. Some patients with stage 4 disease shared that they were ‘talking to the nephrologist about [dialysis] port placement’ or had already placed their permanent ports. Those who shared this experience had refused kidney transplants or were told they were ‘not candidates’.

Patients talked about making health‐related decisions early in their CKD experience. Those with stage 3 disease were focused on dietary and nutritional choices. Those with stage 4 or higher disease shared the same focus on dietary management, but those on dialysis shared more specifics, such as ‘fluid restrictions’ and protein limitations. Those not on dialysis shared nutritional generalities, such as ‘…trying to eat a diabetic type of diet’. Many made nutrition‐based decisions, like undergoing ‘bariatric surgery to lose weight’, driven by a desire to slow the progression of their kidney disease and to control their underlying disease process.

Personal events played a significant role in decision‐making. For example, for those diagnosed with kidney disease at a younger age, the desire to have biological children or commitment to caring for a child influenced their treatment decisions. Older participants shared their own role as caregivers to a spouse or parent impacted decision‐making, especially when the healthcare needs of the family member were accessed as the priority. These dependent care decisions affected multiple aspects of decision‐making, including renal replacement choice, especially kidney transplant over dialysis and medication choice, and prompted evaluation of renal replacement choice when circumstances changed.

#### 3.2.2 Challenges to decision making

##### Not enough information about the pathophysiological process of CKD and available resources

Patient and caregiver participants shared that having enough information was a key factor when making decisions related to their illness. For instance, participants expressed not understanding kidney disease and the extent of damage to their own kidneys. However, participants also shared that they lacked information on the available resources that could assist them in their decision‐making, such as having information about transportation assistance before deciding on renal replacement therapy or knowing ‘anything about transplant’ before starting dialysis. In addition, multiple participants shared that they struggled with dietary restrictions and had little to no information about what foods to choose, though some remarked that they avoided ‘salty foods’ and ate ‘mainly vegetables’. Frustration was voiced as participants spoke about not having the resources they needed to manage their disease before they needed it. For instance, one participant shared ‘if I had known, I think I would've been better prepared … if they would have sat me down and told me what these numbers mean’. This was expressed because this participant had been told her disease was getting ‘a little worse’ and that was supported by giving her ‘my numbers’ but no information was provided that told her what this meant and if she could do anything about it.

##### Co‐morbidities made it difficult to prioritize CKD issues

The presence of co‐morbidities presented a decision‐making challenge. Participants had to balance seeking treatment for a condition(s) that was evaluated as more urgent than their kidney disease while contemplating the potential negative impact those treatments could have on their kidney disease. In addition, sometimes the presence of the condition(s) limited how their kidney disease could be treated, as demonstrated in a male participant's quote, ‘I've not only got kidney disease, I've got heart disease and a hernia that needs to be fixed. But they won't do the hernia surgery and denied me for kidney transplant because of my heart’. Addressed previously, co‐morbidities like hypertension and diabetes were considered when making dietary choices but were not mentioned in the context of those conditions playing a role in decision‐making. Some participants said their kidney disease was the co‐morbidity that they were ‘juggling’ because of the effects of a more predominant disease. A female participant shared that her kidney disease ‘basically came on from damage from chemotherapy…’ and another shared that her kidneys were injured after her experience with liver failure and subsequent liver transplantation. They all share how these competing interests and disease management priorities challenge their decision‐making for kidney disease.

#### 3.2.3 Factors influencing decision‐making

##### Caregivers

Caregivers served in a variety of decision‐support roles to patients. Participants helped patients by ‘being positive and encouraging’, ‘voicing their opinions’ and ‘listening’. In addition, caregivers assisted in decision‐making by managing care after a decision was made. For example, one patient participant commented on her caregiver supporting her weight management—‘…I try to walk, and he walks with me’. Likewise, a married participant shared that her ‘husband … escorts me to each visit so he'll know and understand what I'm dealin’ with on the daily’. Others shared that their caregivers drove them to and from dialysis, arranged for other transportation (usually another family member) and/or helped them with meals and minor house cleaning tasks after a dialysis appointment.

##### Decisions in the medical appointment context

Trust in the clinician was the most mentioned factor influencing decision‐making during medical appointments, followed closely by caregiver support. Clinicians demonstrated trust by making time during the visit to answer questions and provide information, performing thorough examinations, giving feedback and providing easy‐to‐understand explanations. For example, one participant stated: ‘…they was no joke when it comes to … my care. You know, even when I was in the hospital, my doctor came every day, and I loved him. You know it shows he cares’. When participants shared that they trusted their clinician, they asked more questions and had more frequent conversations with the clinician during their appointment or through communication using the healthcare portal. Older patients and those with high school education or less shared a heavier reliance on their clinicians to tell them what they needed and when they needed it, demonstrated by the quote, ‘I don't read to good…they let me know that it's serious, and what I need to do and stuff… I listen to them’.

Caregivers had multiple responsibilities during medical appointments. They served as advocates, information seekers, clarifiers and informants. In the role of informant, patient and caregiver participants shared that caregivers provided information about the patient that the patient might have omitted or did not want to share. For example, a caregiver participant says, ‘Sometimes she [the patient] don't want to tell them everything's that's going on, they ask her how as her appetite … she says, ‘well it's pretty good’ and I'm like, ‘No she don't eat, she needs something to help her eat’. She calls me the warden’. This demonstrates the experience that many shared, advocacy through the role of information sharing. Caregivers worked better in the role of a decision partner when they could be present and hear information ‘firsthand’. With firsthand knowledge, they could help recall discussions, keep track of follow‐ups and answer any patient questions. Caregivers being present in appointments was vital for older patients and those on dialysis. Both stated difficulties remembering everything that was shared in the appointment and always wanted someone with them.

##### Resources

Participants who sought information about their kidney disease mentioned various resources used to help them gather information and support. Primary care providers were approached, especially if the initial diagnosis occurred within that medical practice. Social workers were seen as helpful when gathering resources, but they were only mentioned by those receiving care at a dialysis centre. The other vital resource was other people's stories. Participants shared multiple ways they connected with others via their church, support groups, classes held at a local dialysis centre or through friends to ask questions and understand their kidney disease stories. In these stories, participants, especially patients, shared that they could ‘bounce ideas off each other’, ‘talk about recipes and different things’, and that they found someone who can ‘…understand when you don't have good days’. The general sense was that they could learn from someone's story because something that worked for that individual ‘…might also work for me’ or it could give them something to ask their nephrologist about.

Online resources, specifically those in the Medical Centre's Patient Portal, were used to look up information about various tests, lab values, medications and communication with the medical team. The portal was used by those who shared more experiences about advocating for themselves. When explicitly asked about its use, those who did not mention the portal were unaware of its existence. A few (less than 4) shared that they had received a magazine from the American Association of Kidney Patients. Those who shared this resource were either transplant recipients or had been diagnosed with kidney disease for over 5 years. No one shared a common factor that triggered the magazine's arrival; they just remembered that it arrived at their home.

##### Decision‐making within the context of disease stage

Stage 3 participants' decision‐making focused on dietary management and addressing co‐morbidities that needed more attention. As the disease advanced into stage 4 and higher, decision‐making changed, and participants described deeper thinking about the possibility of ‘living on dialysis’ and how to manage daily life. Participants experiencing end‐stage disease and receiving dialysis shared thoughts regarding the impact of dialysis on everyday life, including needing assistance with household chores, meal preparation and transportation from dialysis appointments. These participants and those who had received transplantation and thus had experienced dialysis previously were the only ones to describe the ‘physical and emotional’ toll of treatment on their everyday life. Discussions about decisions in end‐of‐life occurred with participants experiencing stage 4 or higher disease or if the patient participant had experienced another severe disease even though currently they were in stage 3 CKD.

## 4 DISCUSSION AND CONCLUSION

### 4.1 Discussion

Our findings describe the decision‐making experiences of patients with CKD and their family caregivers from stage 3—end‐stage disease. Patients' and caregivers' reflections and experiences with health‐related decision‐making in CKD demonstrate that decisions are triggered when health declines. Yet, decisions that impact disease progression are being made as early as stage 3. These findings also highlight that decision‐making is made difficult due to a lack of information, the complexity of one's disease state and poor resource utilization. Lastly, caregivers, the structure and nature of the medical appointment, and resources, when used, remove some barriers related to decision‐making by assisting with enhanced communication and greater self‐advocacy.

When viewed holistically, the study findings lend increasing evidence to decision‐making and treatment adherence literature, demonstrating a direct connection to knowledge, resource utilization and positive provider factors that can improve decision conflict and enhance trust for future decisions in CKD and other serious illnesses.^13^, ^37^, ^44^, ^45^ Supportive caregivers and a trusted relationship with their clinician were presented as mechanisms supporting decision‐making. While this supports decision‐making literature, highlighting that discussions should be bidirectional, this study emphasizes the importance of including caregivers in the conversation.^46^ In addition, similar findings regarding engaged caregivers can be found in CKD,^47^, ^48^, ^49^ yet these studies more often detail the benefits of engaged caregiving during dialysis and lack of focus on caregiver influences throughout illness.^50^, ^51^, ^52^ In contrast, our findings illustrate the benefits of engaged caregiving upstream of advanced disease. This finding lends additional support to emphasizing the importance of appreciating and exploring the complex social networks that affect decision‐making and how decisions are reached due to cognitive processes involving more than one individual.^28^ Overall, there was a clear description that the medical appointment with a trusted clinician is where the majority of patients gather the support, knowledge, and opinions needed to make decisions for disease management.^53^, ^54^, ^55^ In addition, the protective nature of having access to multiple resources demonstrated a solid connection to earlier renal replacement decisions, which equated to earlier access to transplant, greater access to support resources and more advocacy for self in medical appointments. Differences in decision‐making between Black and White respondents were not prominent. The decision‐making description provided reflects a common shared experience.

There are notable strengths and limitations to this study. A strength is the diverse representation of disease stage, age and transplantation status, which means that decision‐making perspectives along the trajectory of illness are represented. In addition, interviewing caregivers provided a more holistic view of the decision‐making experience and what influences the process. This is important because caregivers are intimately involved in healthcare decision‐making, and understanding this influence is vital to enhancing decision‐making support and self‐management.^14^, ^51^, ^52^ This reflection of decision‐making supports a relationally‐centred and not patient‐centred approach, especially when dealing with advanced CKD decisions.^56^

Limitations include single‐site recruitment, limiting the representation of views where nephrology services may differ. In addition, there were no interviews to capture clinical perspectives on decision‐making, which can be remedied with a follow‐up study. Lastly, there is no representation of other racial or ethnic minorities (i.e., Hispanic/Latino individuals) and those identifying as Lesbian, Gay, Bisexual, Transsexual or Queer+. Lacking these perspectives on decision‐making limits this work's generalizability and will need to be addressed in future works to ensure intervention development can meet the complex needs of these populations.

### 4.2 Conclusion

From our results, four major implications emerged for intervention development regarding the decision‐making experience of patients and families within a CKD context. First, our findings support that intervention should occur earlier in the disease.^54^ To facilitate patients being able to build more knowledge, interventions should identify additional members of the healthcare team who can support decision‐making or enable access to resources resulting from the decisions being made. These allied professionals (i.e., dietary consult and social work) are available through clinical referral and are covered under most public and private insurance plans, and can be accessed at least once a year for continued support. Second, interventions must enable patients and caregivers to engage in decision‐making.^5^ Decision support interventions must do more than point to resources; these interventions must empower dyads to ask questions, seek answers and request what they need to manage their disease process as activated and engaged healthcare team members.^37^, ^57^ Third, we suggest that interventions include methods that enhance communication skills both patient‐to‐clinician and patient‐to‐caregiver. While patient‐to‐clinician is best represented in the literature,^58^, ^59^, ^60^ patient‐to‐caregiver communication is also important. Our findings demonstrated that patients and caregivers are talking, and targeted communication training is needed so that the dyad can communicate effectively around values, treatment desires and social support needed. These conversations can be a strong catalyst for better resource utilization. Fourth, we strongly recommend that these interventions review decision aids that could help the patient and caregiver explore concepts related to renal replacement on their own, allowing them to access their values and understand how their values align with the available options.^35^, ^38^, ^54^

### 4.3 Practice implications

In conclusion, this study provides compelling evidence that the majority of patients and caregivers face difficulty when making health‐related decisions throughout the disease trajectory. In addition, their experiences highlight that there are resources, including the support of an attentive caregiver and trusted clinician, which can be utilized to improve decision‐making; yet not all dyads know these resources are present or how to gain access. Therefore, this work is critical in future intervention development. Based on these results, we hypothesize that early intervention is critical, especially those that encompass training members of the decision partnership team, patient, caregiver(s) and clinician, to function as engaged members in shared decision‐making. Overall, the goal should be to support patients and caregivers from diagnosis to death so that no patient feels rudderless, as if they have to live their life ‘stuck in the middle’ where they lack access to critical support that could help them live a higher quality of life.

## AUTHOR CONTRIBUTIONS

Shena Gazaway and James N. Odom developed the research idea and study design. Shena Gazaway and Tamara Nix‐Parker collected data and performed data analysis. Rachel Wells and Claretha Lyas wrote sections and were critical to the editing of the manuscript for final submission. Orlando Gutierrez and James N. Odom both served as mentors for study and manuscript development. All authors contributed to the study implementation, design and preparation of the manuscript.

## CONFLICT OF INTEREST STATEMENT

The authors declare no conflict of interest.

## ETHICS STATEMENT

This study was approved by the University of Alabama at the Birmingham Institutional Review Board (IRB‐300008301) and was conducted in accordance with the 1964 Declaration of Helsinki and its later amendments. Informed consent was collected from all participants.

## Data Availability

Research data are not shared.
